# Farmland boundary extraction based on the AttMobile-DeeplabV3+ network and least squares fitting of straight lines

**DOI:** 10.3389/fpls.2023.1228590

**Published:** 2023-08-18

**Authors:** Hao Lu, Hao Wang, Zhifeng Ma, Yaxin Ren, Weiqiang Fu, Yongchao Shan, Shupeng Hu, Guangqiang Zhang, Zhijun Meng

**Affiliations:** ^1^ Intelligent Equipment Research Center, Beijing Academy of Agriculture and Forestry Sciences, Beijing, China; ^2^ State Key Laboratory of Intelligent Agricultural Power Equipment, Beijing, China; ^3^ National Engineering Research Center of Intelligent Equipment for Agriculture, Beijing, China; ^4^ School of Integrated Circults and Electonics, Beijing Institute of Technology, Beijing, China; ^5^ Beijing Research Center for Information Technology in Agriculture, Beijing Academy of Agriculture and Forestry Sciences, Beijing, China

**Keywords:** UAV remote sensing, farmland boundary extraction, semantic segmentation, DeeplabV3+, linear fitting

## Abstract

The rapid extraction of farmland boundaries is key to implementing autonomous operation of agricultural machinery. This study addresses the issue of incomplete farmland boundary segmentation in existing methods, proposing a method for obtaining farmland boundaries based on unmanned aerial vehicle (UAV) remote sensing images. The method is divided into two steps: boundary image acquisition and boundary line fitting. To acquire the boundary image, an improved semantic segmentation network, AttMobile-DeeplabV3+, is designed. Subsequently, a boundary tracing function is used to track the boundaries of the binary image. Lastly, the least squares method is used to obtain the fitted boundary line. The paper validates the method through experiments on both crop-covered and non-crop-covered farmland. Experimental results show that on crop-covered and non-crop-covered farmland, the network’s intersection over union (IoU) is 93.25% and 93.14%, respectively; the pixel accuracy (PA) for crop-covered farmland is 96.62%. The average vertical error and average angular error of the extracted boundary line are 0.039 and 1.473°, respectively. This research provides substantial and accurate data support, offering technical assistance for the positioning and path planning of autonomous agricultural machinery.

## 1 Introduction

Autonomous unmanned operation technology for agricultural machinery is one of the vital technologies for realizing precision agriculture (Wang and Noguchi, 2016; Shafi et al., 2019). This technology has been widely used in tasks such as ploughing, sowing, harvesting, and fertilizing, significantly reducing the consumption of human labor and resources (Wang et al., 2021b; Yang et al., 2022; Bai et al., 2023). Many researchers have found that the accurate acquisition of farmland boundaries is the basis for autonomous and safe operation of farm machinery in the field (Li et al., 2020; Kim et al., 2021; Wang et al., 2021c; Li et al., 2023a; Yu et al., 2023). However, relying on field staff to go deep into the fields to collect data has low work efficiency and is time-consuming (Ingram, 2018). With the development of equipment such as vehicle-mounted LiDAR, the technology for ground vehicles to obtain farmland boundaries in real time has become relatively mature (Xu et al., 2019; Nehme et al., 2021). However, such methods are difficult to apply in complex farmland scenarios. For example, there are many irregular obstacles in the field, areas that cannot be travelled by farm machinery, and in some cases, “farmland with crops” and “farmland without crops” are similar in color and cannot be clearly distinguished. In recent years, rapid segmentation technology based on remote sensing images has provided a low-cost and efficient method to obtain boundaries. Remote sensing image acquisition work is mainly based on satellite remote sensing (Marshall et al., 2019; Segarra et al., 2020; Waldner and Diakogiannis, 2020). However, satellite images encounter problems such as unclear distinction in fine structure areas and delayed imaging. Meanwhile, unmanned aerial vehicle (UAV) remote sensing is rapidly developing and gradually being incorporated into various agricultural applications (Xu et al., 2020; Wang et al., 2021a). The resolution of UAV remote sensing can reach decimeter levels, not only accurately identifying regional boundaries but also creating favorable conditions for identifying farm lanes, ditches, and ridges. Therefore, this paper adopts UAV remote sensing technology to acquire farmland images.

High-resolution farmland remote sensing images bring abundant geospatial information, which consequently increases the difficulty of extracting farmland boundaries (Xia et al., 2019). Wang et al. (2021c) developed a Grabcut-based farmland segmentation system and detected centimeter-level accuracy field boundary from the UAV imagery. Traditional image processing methods have limited effectiveness when it comes to identifying high-resolution UAV remote sensing images (Jeon et al., 2021). To obtain more reliable and accurate information on farmland plot distribution, researchers have used fully convolutional neural networks (FCNs) to extract deep semantic features, achieving end-to-end learning (Masoud et al., 2019). Wang et al. (2019) demonstrated that the performance of FCNs in processing spatial features of images significantly surpassed traditional image processing methods.

While existing semantic segmentation networks have made certain advancements in recognizing UAV farmland images, obtaining a complete boundary segmentation image remains a challenge. DeeplabV3+ was proposed by Google researchers in 2018; this network, based on spatial pyramid pooling technology and integrating the advantages of multiple networks, has achieved excellent results in semantic segmentation tasks (Chen et al., 2018). In the realm of agricultural domain, many improved DeeplabV3+ algorithms have made considerable progress, as showcased in the research of Lu et al. (2022) and Li et al. (2023b). Therefore, this paper considers improving the DeeplabV3+ network to address the aforementioned issues.

After segmentation, it is necessary to extract the boundary lines from the farmland images. Common line extraction methods include the Hough Transform (HT) (Koc-San et al., 2018; Chen et al., 2021), the Random Sample Consensus (RANSAC) algorithm (Ji et al., 2021; Kok et al., 2023), and the Least Squares (LS) method (Acharya et al., 2022; Ma et al., 2022). The HT can detect all lines in the image in one go, boasting high computational efficiency. However, the detection results are constrained by factors such as time, space, and image noise. The RANSAC algorithm is not affected by noise or outliers in the dataset, but the complexity of setting parameters such as iteration and thresholds results in lower computational efficiency. The advantage of the LS method is its simplicity and intuitiveness. Compared to the other two methods, it does not require manual parameter setting and it offers higher computational efficiency. Therefore, this study chooses the LS method for farmland boundary line extraction.

This paper proposes a method for segmenting farmland regions and extracting boundary lines based on drone remote sensing. The main contributions of this work are as follows:

1. To address the issue of incomplete farmland boundary segmentation, this paper proposes an improved DeeplabV3+ network, which uses MobileNetV2 as the backbone network to reduce computational load. With the inclusion of the Convolutional Block Attention Module (CBAM) attention mechanism module into the backbone network, the use of shallow network detail information can be fully exploited, enhancing the network’s precision in recognizing farmland details. Adjustments to the Atrous Spatial Pyramid Pooling (ASPP) module have also been made by adding a Strip Pooling Layer (SPL), which improves the network’s accuracy and efficiency in recognizing long-range dependency features. This further enhances the precision of farmland edge segmentation in UAV images.
2. Based on the output results of the aforementioned semantic segmentation network, a farmland boundary line extraction algorithm has been designed. This algorithm segments the two types of farmland areas and non-farmland areas in the image, then tracks the binary image boundary using MATLAB’s bwboundaries boundary tracking function to obtain a boundary coordinate dataset. Finally, the method of least squares is used to fit the boundary line, providing boundary information for autonomous unmanned agricultural machinery, and laying the data foundation for subsequent boundary coordinate extraction.

## 2 Materials and methods

### 2.1 Field test and image acquisition

#### 2.1.1 Data acquisition

**Figure 1:**
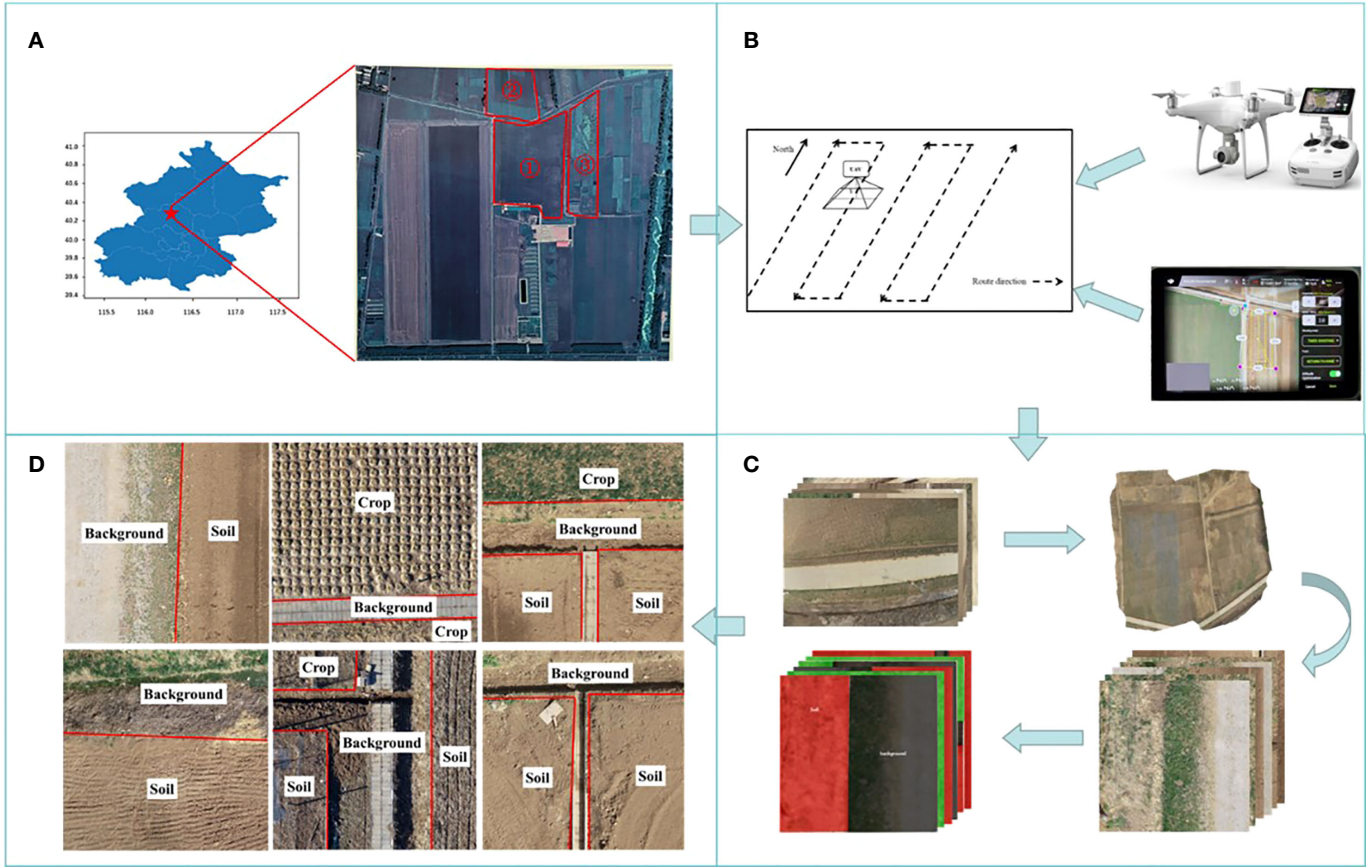
Workflow of the data acquisition phase. **(A)** Experimental area **(B)** UAV route setting; **(C)** UAV capture of original image set; **(D)** Label element,Label information for each area is marked in white.

Data collection was conducted from March to April 2022, at the National Precision Agriculture Demonstration Base (Beijing, China), which covers an area of 2,500 mu. As shown in **Figure 1A**, we selected three high-standard farmland plots from the base for data collection. For drone image collection, it should be done during clear weather, with an average wind speed of less than 5 m/s, and abundant daylight. To ensure the quality of the aerial photography, shooting times were selected between 10:00–12:00 and 13:00–16:00.

The data collection platform is the consumer-grade drone DJI Phantom 4 PTK, which is equipped with a RedGreenBlue (RGB) camera and a built-in Real Time Kinematic-Global Navigation Satellite System (RTK-GNSS). The effective pixel count of the camera is 20 million, with a maximum image resolution of 5,472×3,078 pixels. The RTK-GNSS can provide a horizontal positioning accuracy of 1 cm + 1 ppm (Recipe Manager System, RMS) and a vertical positioning accuracy of 1.5 cm + 1 ppm (RMS). The drone autonomously operates along the flight path specified in **Figure 1B**, with a flight speed set at 2 m/s and a flight altitude of 25 m. Owing to the wide-angle lens used by the drone, the generated images have very obvious radial distortion (Kang et al., 2021), which can affect the accuracy of image edge segmentation. To address image distortion as much as possible, the flight path and longitudinal overlap rates were set to 70% in reference to related technical specifications (Ma and Zhang, 2022) to alleviate distortion after image stitching. The DJI Terra software was then used to stitch together the obtained farmland images.

#### 2.1.2 Preparation of the dataset

First, data processing is carried out to obtain accurate and reliable base image data. The stitched images are in the Tagged Image File Format (TIFF). TIFF is divided into the “coordinate tag” module and the “RGB channel data” module, which offer high clarity, high resolution, and an abundance of information. These features are advantageous for subsequent random cropping and dataset division. In order to preserve the geographic coordinate information of the images and meet the accuracy requirements for extracting farmland boundaries, the original image data are randomly cropped into PNG images with a pixel size of 1024×1024. Each image is named after the pixel coordinates of the first pixel in the image. To further refine the data samples, experimental images with low resolution are eliminated, yielding a total of 7,977 images.

Next, a dataset is built to meet the requirements of the experiment. The process is as follows: we use the open-source annotation tool LabelMe to semantically annotate UAV remote sensing images following the style of the Pascal VOC2007 dataset, thus creating a standard semantic dataset. Each image is ensured to have a corresponding label file. During the annotation process, we noticed that some image elements have a large span in color space, which could lead to misidentification by the network during image segmentation, thereby decreasing the overall recognition accuracy. As such, this study analyzed the definition of boundary positions, taking into account that an excess of label elements might interfere with the precision of farmland boundary recognition. The remaining interfering elements were consolidated into a category labeled “Background”. To ensure the completeness, reliability, and practicality of the dataset, it was further refined. Now, the label elements are divided into three categories: “Crop”, “Soil”, and “Background”. Here, “Crop” refers to farmland with crops, while “Soil” denotes farmland without crops. The processed dataset contains a total of 6,386 images, with 3,285 “Crop” labels and 3,356 “Soil” labels. The images are randomly divided into training and validation sets at a 9:1 ratio. **Figure 1C** displays the process of dataset creation, while **Figure 1D** presents the various labeled elements in the dataset, which contains several environmental scenarios: the case where only one label type exists in the image bordering the background and the case where multiple label types exist in the image bordering the background.

### 2.2 Construction of the farmland extraction model based on the AttMobile-DeeplabV3+ model

#### 2.2.1 AttMobile-DeeplabV3+ model

**Figure 2:**
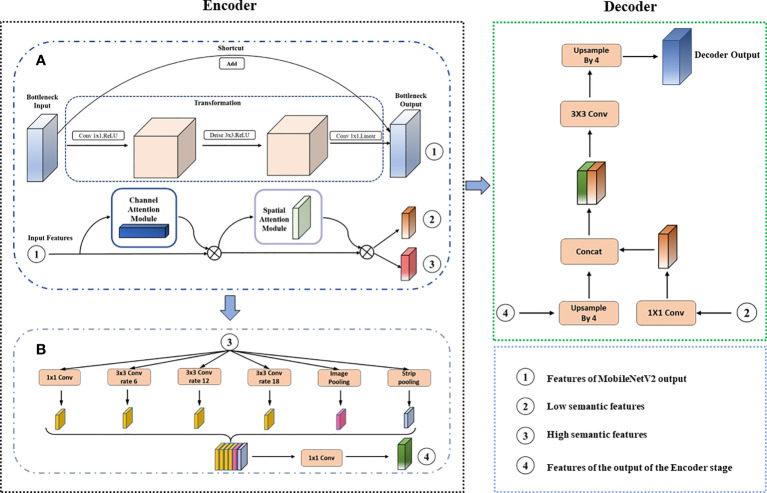
**(A, B)** AttMobile-DeeplabV3+ model structure diagram.

Before detecting the farmland boundary lines, it is necessary to first obtain images containing farmland boundary information and separate the farmland areas from the non-farmland areas. In outdoor real farmland environments, the demarcation line of farmland is not clear and difficult to distinguish. Moreover, most of the boundaries in farmland images have a long straight line-type distribution with long-range dependence, as shown in **Figure 1D**. For certain visually similar classes to be identified, it is somewhat challenging to use classical semantic segmentation networks. To address these issues, we make improvements to the existing DeeplabV3+ network and propose an enhanced DeeplabV3+ network using MoblieNetV2 as the backbone network, with the addition of CBAM. This enhanced network is referred to as AttMobile-DeeplabV3+. The network structure is shown in **Figure 2**.

The AttMobile-DeeplabV3+ maintains the same encoder–decoder structure as DeeplabV3+, with improvements made to the backbone network. The traditional DeeplabV3+ network uses the Xception network as its backbone, which employs Depth-wise Separable Convolution (Howard et al., 2017), enhancing the network performance. However, because of the complex computation process, the network convergence speed is relatively slow. Hence, AttMobile-DeeplabV3+ adopts MobileNetV2 as the backbone network, introducing the Convolutional Block Attention Module to select beneficial features and suppress irrelevant ones, thereby improving the overall segmentation precision. Additionally, it makes improvements to the ASPP module, incorporating a pooling operation (SPL) to enhance the extraction accuracy of long-range dependencies.

The workflow of AttMobile-DeeplabV3+ is approximately as follows: the image under test is input into the network, where feature extraction and attention enhancement are performed through operations such as convolution and pooling. Then, the features extracted from the encoder and decoder are fused, the channel numbers of the feature layer are adjusted, and the sizes of the input and output images are matched through upsampling operations, eventually yielding the prediction result.

(1) Design of Attention-MobileNetV2 Network

Attention-MobileNetV2 is a crucial improvement part in this study, with its primary structure illustrated in **Figure 2A**. MobileNetV2 splits depthwise separable convolution into depthwise convolution (Depth-wise, DW) and pointwise convolution (Point-wise, PW), which enhances the convolutional efficiency and reduces the computational time of the network. Additionally, the core of this network utilizes Inverted Residuals for feature extraction. Compared to traditional convolutional structures, depthwise separable convolution requires fewer adjustable parameters and less computation, thus mitigating the risk of network overfitting.

**Figure 3:**
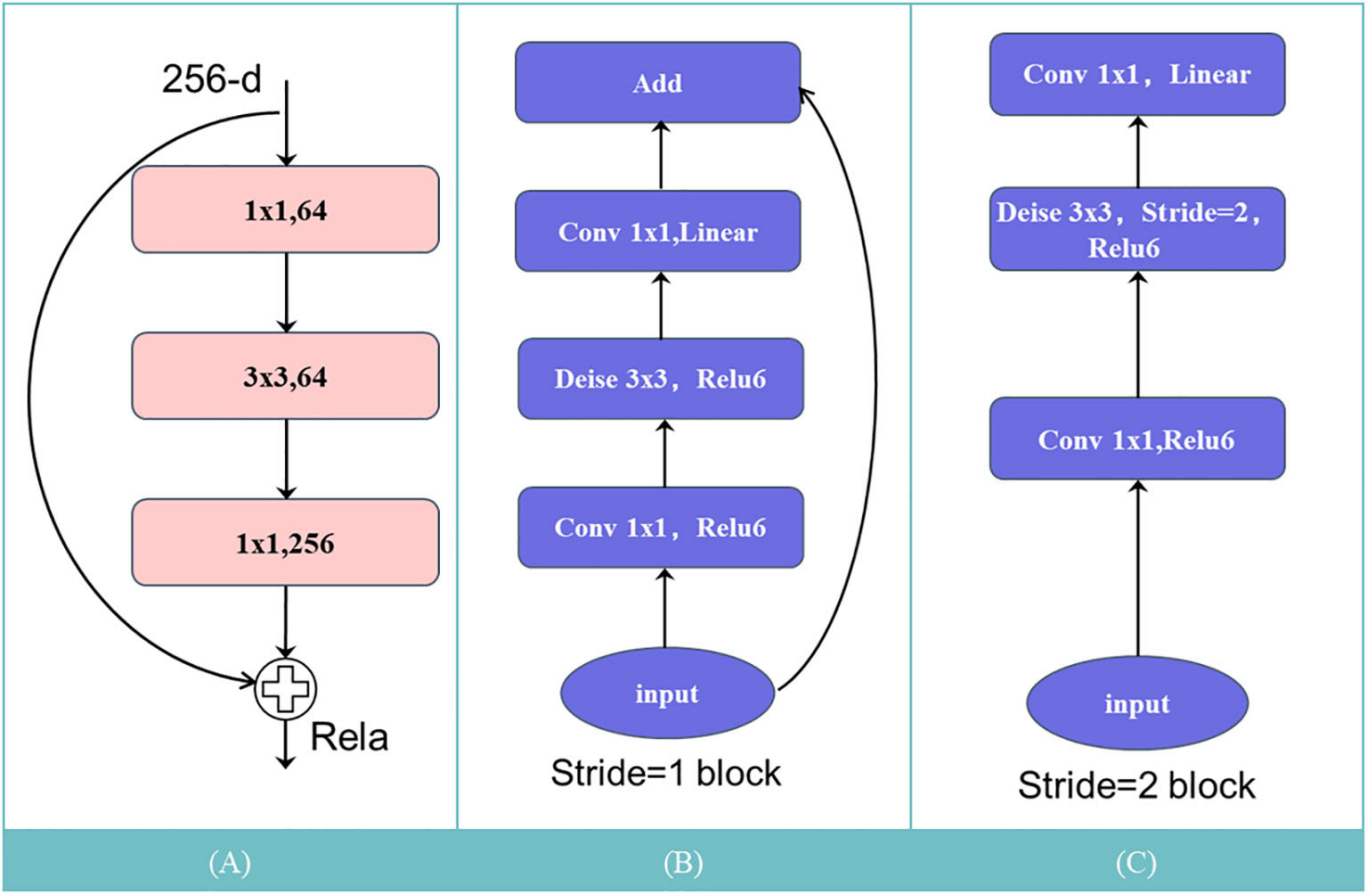
Inversion residual: **(A)** Residual structure; **(B)** Inverse residual structure (with shortcut); **(C)** Inverse residual structure (no shortcut).

The Inverted Residuals structure of MobileNetV2, an improvement over the residual structure of MobileNetV1 (Sandler et al., 2018), optimizes the latter’s inherent characteristics. Traditional residual structures initially decrease the channel count of feature maps through a 1 × 1 convolution layer, perform a 3 × 3 convolution, then expand the channel count using another 1 × 1 convolution layer, finally adding it to the input feature map as depicted in **Figure 3A**. Conversely, the Inverted Residuals structure of MobileNetV2 first expands the channel count of input feature maps using a 1 × 1 convolution layer, then applies depthwise separable convolution as shown in **Figure 3B**. In the end, to prevent excessive information loss, Linear Bottleneck is utilized to decrease channel count, replacing the original non-linear activation transformation. This structural improvement significantly boosts the network’s computational efficiency and accuracy, proving to be more suitable for segmentation tasks under limited GPU power.

While enhancing the speed of network segmentation tasks and alleviating the GPU’s workload, improving the network structure inevitably impacts segmentation accuracy to a certain extent. Furthermore, given the complexity of agricultural scenes, parcel edges often encounter issues of misclassification or omissions. As the research shows (Woo et al., 2018), the ability of a network to focus on crucial information improves when a CBAM is added, with more features of the object to be recognized being covered, thereby enhancing the final object identification accuracy. This paper introduces the convolutional block attention module into MobileNetV2, the structure of which is shown in **Figure 1B**. This module, including a channel attention mechanism (CAM) module and a spatial attention mechanism (SAM) module, effectively addresses the aforementioned issues.

The process of generating attention through the CBAM module is shown in Equations (1) and (2).


(1) $${\text{F}}^{\prime }={\text{M}}_{\text{c}}(\text{F})⊗\text{F}$$

(2) $${\text{F}}^{\prime \prime }={\text{M}}_{\text{S}}({\text{F}}^{\prime })⊗{\text{F}}^{\prime }$$

In equations, $\text{F}\in {\text{R}}^{\text{C}\times \text{H}\times \text{W}}$ represents the output weight data from the backbone network, the expression for the CAM module is ${\text{M}}_{\text{C}}\in {\text{R}}^{\text{C}\times 1\times 1}$ , and the expression for the SAM module is ${\text{M}}_{\text{S}}\in {\text{R}}^{1\times \text{H}\times \text{W}}$ . At this point, the output weight data for CAM is 1×1×C and that for SAM is 2×H×W. F’ is the result of the channel attention output. After merging F’ with the spatial attention weights, the final output result F” of the CBAM module is obtained.

(2) Improved Atrous Spatial Pyramid Pooling

**Figure 4:**
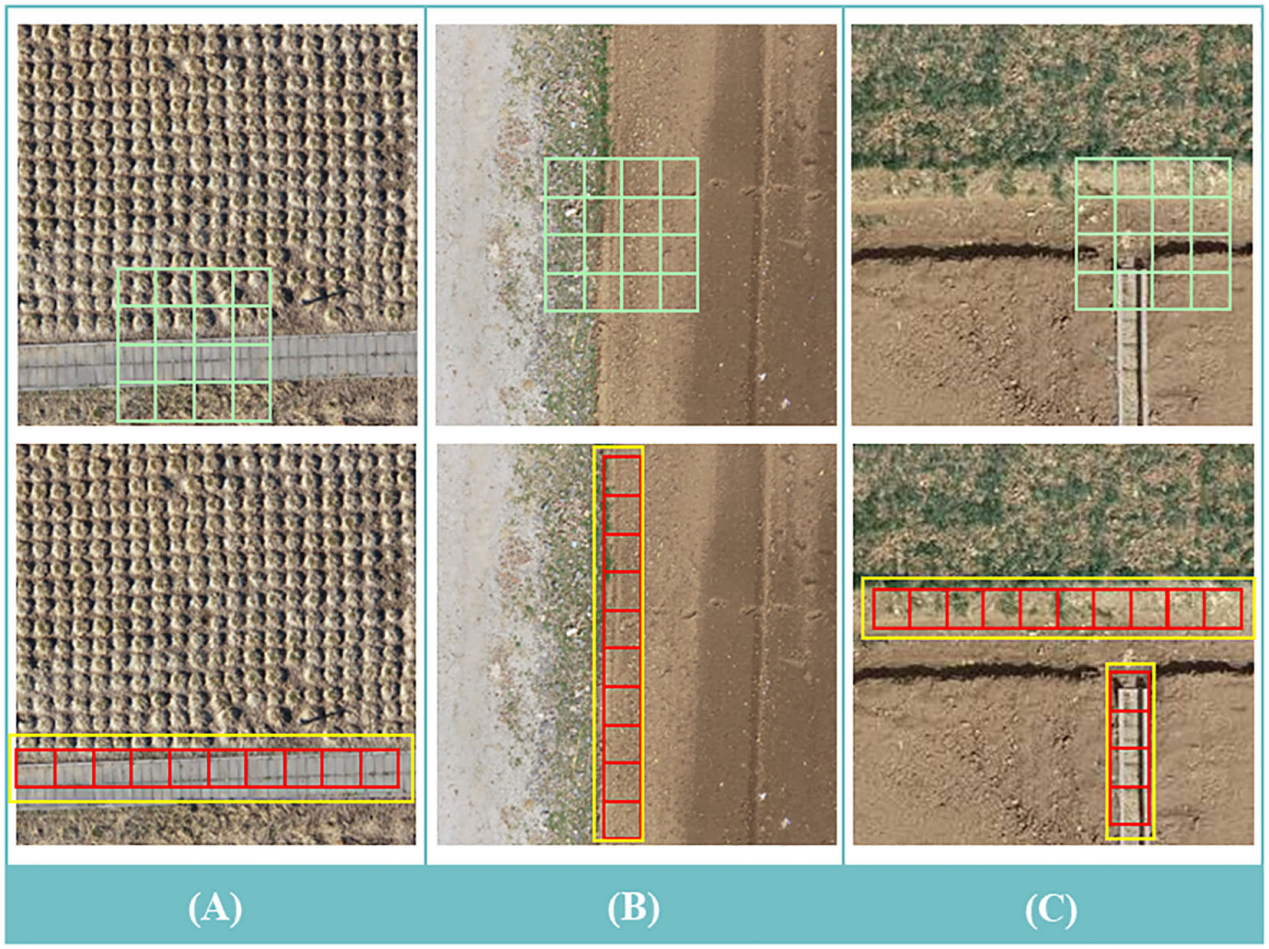
**(A–C)** An illustration of how strip pooling and traditional spatial pooling work differently in scene resolution. From top to bottom: traditional spatial pooling; strip pooling. As shown in the top row, strip pooling has a strip kernel (red grid) compared to traditional spatial pooling (green grid), and therefore captures remote dependencies between discrete distributions of regions (yellow bounding boxes).

The ASPP samples the input feature map in parallel with the convolution of voids at different sampling rates; The resulting outputs are then concat together and a 1×1 convolution is applied to reduce the channel number to the desired value. However, both atrous convolution and pooling operate within a square window on the input feature map, which limits their flexibility in capturing anisotropy and context in real-world scenarios. In the farmland scenes studied in this research, target objects often have long-range rectangular structures, such as the farm paths shown in **Figure 4A**, the farm edges in **Figure 4B**, and the irrigation ditches in **Figure 4C**. The use of square pooling windows inevitably includes interfering information from unrelated areas.

Research suggests (Hou et al., 2020) that strip pooling layers can utilize global pooling to optimize the recognition of target features and overcome long-range dependencies. This layer consists of two paths that capture long-range context feature information along the horizontal and vertical spatial dimensions, respectively. For each spatial position in the pooling box, it encodes the global horizontal and vertical information and uses this encoding to balance its own weight for feature refinement, effectively expanding the receptive field of the current module. Using strip pooling can handle more complex scenarios and improve segmentation results, as illustrated in **Figure 4**.

**Figure 5:**
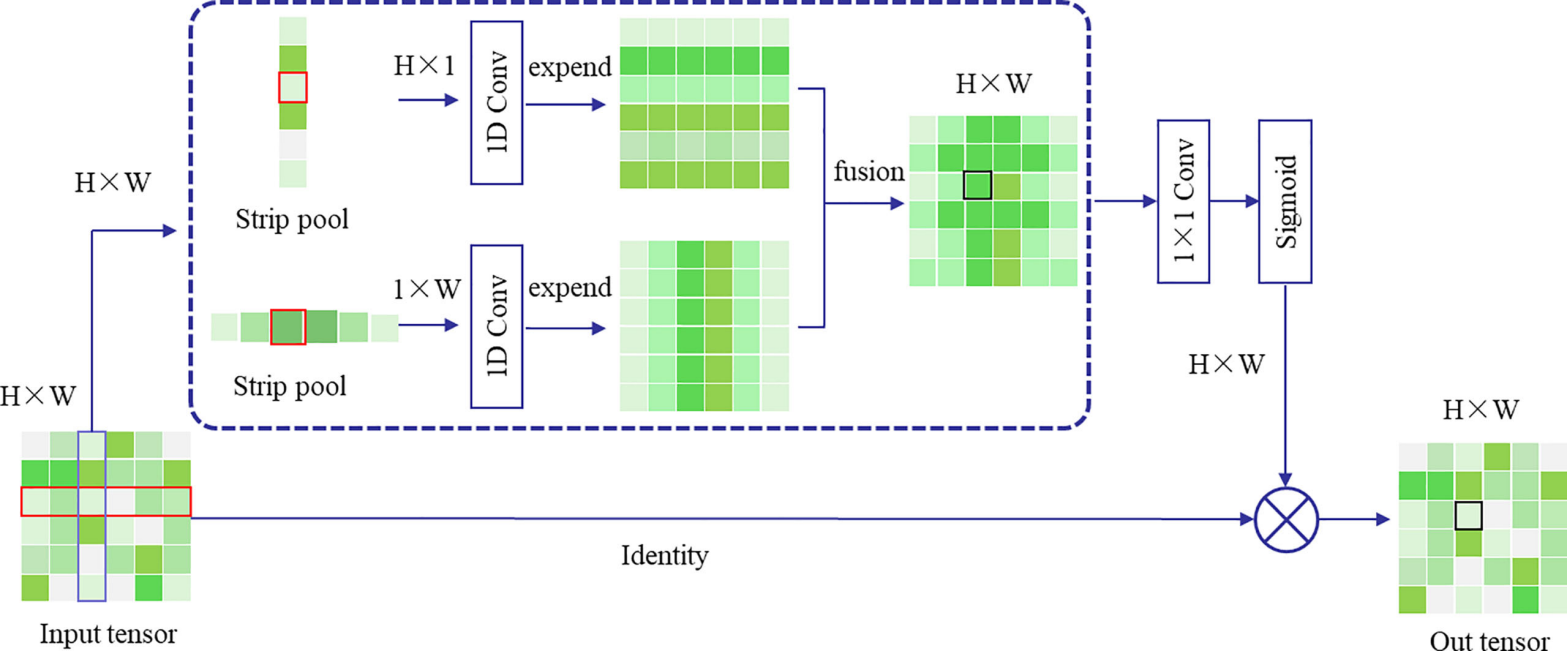
Strip pooling Model.

**Figure 5** illustrates the operation process of the strip pooling layers. Its working principle is as follows: firstly, a feature map F of dimensions C×H×W is inputted. After horizontal and vertical strip pooling, this feature map transforms into dimensions of H×1 and 1×W, respectively. The output values of the pooling operation are the mean values of the elements within the pooling kernel. Then, two feature maps are expanded along the left–right and up–down dimensions, respectively, using a 1×D convolution until they are the same size. The summation of the corresponding positions in the expanded feature maps yields a new feature map of H×W. Following this, the feature map is processed through a 1×1 convolution and a sigmoid function. The resultant feature map is then element-wise multiplied with the original input map to yield the final result.

In this study, the incorporation of Strip Pooling for network optimization can improve the accuracy of farmland plot boundary recognition. By integrating Strip Pooling as an additional pooling layer within the ASPP, the deep features outputted by Attention-MobileNetV2 can be effectively extracted. **Figure 2B** illustrates the overall operating structure of the enhanced ASPP module.

#### 2.2.2 Experimental parameters and network training

The experiment was conducted on a server at the Beijing Supercomputing Cloud Center. The hardware environment comprises a Windows server-grade CPU, the Intel(R) Platinum 8260, with 128 GB of running memory, and two Tesla T4-16 GB computational cards. CUDA version 11.2 was employed. The programming was compiled using Python, while the excellent PyTorch was adopted as the deep learning framework. To ensure the full utilization of the computational capabilities of both graphics cards during the training process, the Data Parallel (DP) module from PyTorch was integrated for distributed training.

The related parameter settings are as follows: the batch size is set to 8, training without freezing the network is employed, the initial learning rate is set to 5e^−4^, and the minimum learning rate is set to 5e^−6^. Cosine annealing (Cos) (Loshchilov and Hutter, 2016) is introduced as the learning rate adjustment strategy in this study to ensure good convergence of the network while preventing overfitting. To adapt to the rate of learning changes, we incorporate the Adaptive Moment Estimation (Adam) optimizer (Kingma and Ba, 2014), setting momentum to 0.9. The Dice_Loss function is chosen as the loss function to overcome issues of imbalance between foreground and background.


(3) $$\text{Dice\_Loss}=1-\frac{2\sum_{\text{i}=1}^{\text{N}}{\text{p}}_{\text{i}}{\text{g}}_{\text{i}}}{\sum_{\text{i}=1}^{\text{N}}{\;}_{\text{pi}}^{2}+\sum_{\text{i}=1}^{\text{N}}{\;}_{\text{gi}}^{2}}$$

In the formula, *N* represents the total number of pixels, *p_i_* is the value of the *i*th pixel predicted by the network, and *g_i_* is the value of the *i*th pixel in the actual label. The range of Dice_Loss is between 0 and 1.

#### 2.2.3 Model evaluation metrics

To compare the actual results and performance of the segmentation algorithm and compare it with other algorithms, this study uses the network’s accuracy on the test set, mean pixel accuracy (mPA), and mean intersection over union (mIoU) to measure the network’s performance. Meanwhile, the number of network parameters (Parameters) and the average inference time on a single dataset image are used to measure the network’s complexity.

mPA is a metric that measures the accuracy of each class in the segmentation task. PA calculates the ratio between the number of correctly classified pixels and the total number of pixels for each class. PA balances the impact of different objects in the image on the segmentation metric and provides a more realistic reflection of the segmentation results for each class. mPA is then calculated by averaging the PA values for all classes. mIoU is a metric that measures the overlap between the ground truth and predicted segmentation masks for each class. It calculates the ratio of the intersection to the union of the predicted and ground truth masks for each class, and then averages these values across all classes. mIoU is computed by calculating the IoU for each class and then averaging the results. Assuming there are *k* + 1 classes, including *k* object classes and one background class, *p_ij_* represents the number of pixels predicted as class *j* while they actually belong to class *i*. The formulas for PA, mIoU, and mPA are shown in Equations (4)–(6).


(4) $$\text{PA}=\frac{\sum_{\text{i}=0}^{\text{k}}{\text{p}}_{\text{ii}}}{\sum_{\text{i}=0}^{\text{k}}\sum_{\text{j}=0}^{\text{k}}{\text{p}}_{\text{ij}}}$$

(5) $$\text{mPA}=\frac{1}{\text{k}+1}\sum_{\text{i}=0}^{\text{k}}\frac{{\text{p}}_{\text{ii}}}{\sum_{\text{j}=0}^{\text{k}}{\text{p}}_{\text{ij}}}$$

(6) $$\text{mIoU}=\frac{1}{\text{k}+1}\sum_{\text{i}=0}^{\text{k}}\frac{{\text{p}}_{\text{ij}}}{\sum_{\text{j}=0}^{\text{k}}{\text{p}}_{\text{ij}}+\sum_{\text{j}=0}^{\text{k}}{\text{p}}_{\text{ji}}-{\text{p}}_{\text{ii}}}$$

Inference time (computing time) is the average time taken by the network to make inferences about the images on the test set.

Number of parameters: The total amount of code that needs to be run in total, generally used to quantify the complexity of the network.

#### 2.3 Farmland boundary extraction method

**Figure 6:**
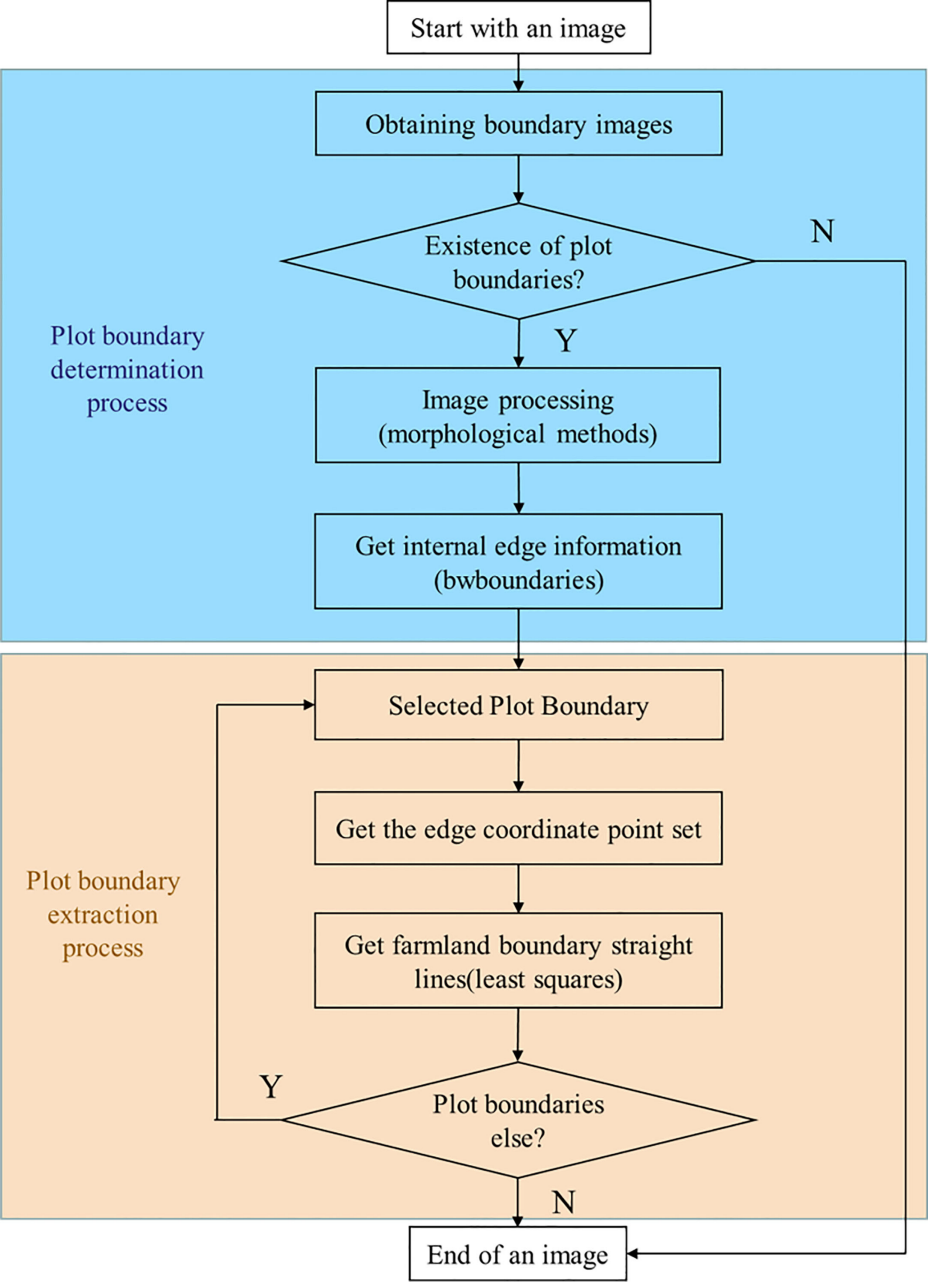
Farmland boundary extraction algorithm workflow diagram.

Using the farmland boundaries extraction network established in *Section 2.2*, the image is segmented into farmland regions and non-farmland regions. To extract farmland boundary lines, we propose a method based on bwboundaries edge detection and least squares fitting to generate straight lines. **Figure 6** shows the complete flowchart of the algorithm, which consists of two main components: coordinate extraction of the plot boundaries and prediction of the farmland boundaries. The following will provide a detailed explanation of these components.

#### 2.3.1 Plot boundary determination and extraction of its coordinates

**Figure 7:**
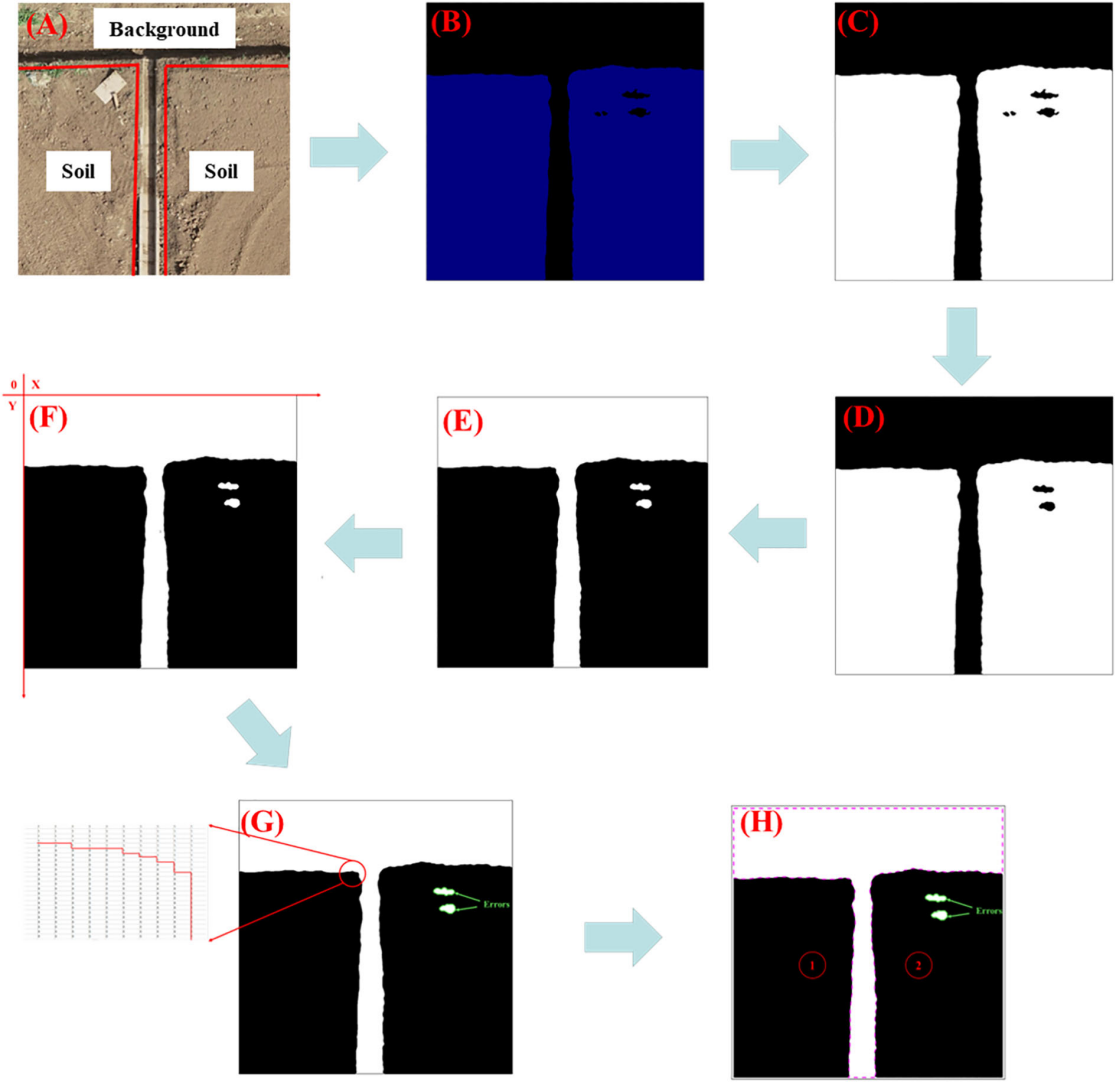
Diagram of the farmland boundary coordinate extraction exercise. The purple dotted area is the boundary area: **(A)** Original image containing parcel information; **(B)** segmented image; **(C)** binary image; **(D)** Image after morphological processing; **(E)** inverted binary image; **(F)** adding a Cartesian coordinate system to the image; **(G)** the regional determination process; **(H)** image after parcel classification.

Taking **Figure 7** as an example, **Figure 7A** shows an image of an agricultural field: it consists of two farmlands without crops and a field canal, and **Figure 7B** is the predicted result. First, the segmented image is converted into a grayscale image and then binarized. Next, we use morphological operations on the image: first, erode the test image, then dilate it to remove tiny isolated noise points, obtaining results as shown in **Figure 7D**. Subsequently, based on the binarized image, we list a binary matrix and convert the binary values from black and white, and this aids later edge extraction. A Cartesian coordinate system is established in the image (with the *x*-axis as the horizontal axis, the *y*-axis as the vertical axis, and the center of the first pixel from the top left of the image as the origin; each pixel center represents a coordinate point).

By iterating through the black pixels in the image, the number of internal plots within the image is determined, while excluding regions with a low number of pixels as they are likely to be misidentified areas, as shown in **Figure 7G**. Once the plots are identified, the coordinate ranges for each region are defined based on the established Cartesian coordinate system. Finally, the bwboundaries function is used to obtain the boundary regions for each farmland, shown as purple dashed lines in **Figure 7H**. Additionally, the coordinate point sets for the intersections between the black and white regions are obtained.

#### 2.3.2 Fitting boundary line

**Figure 8:**
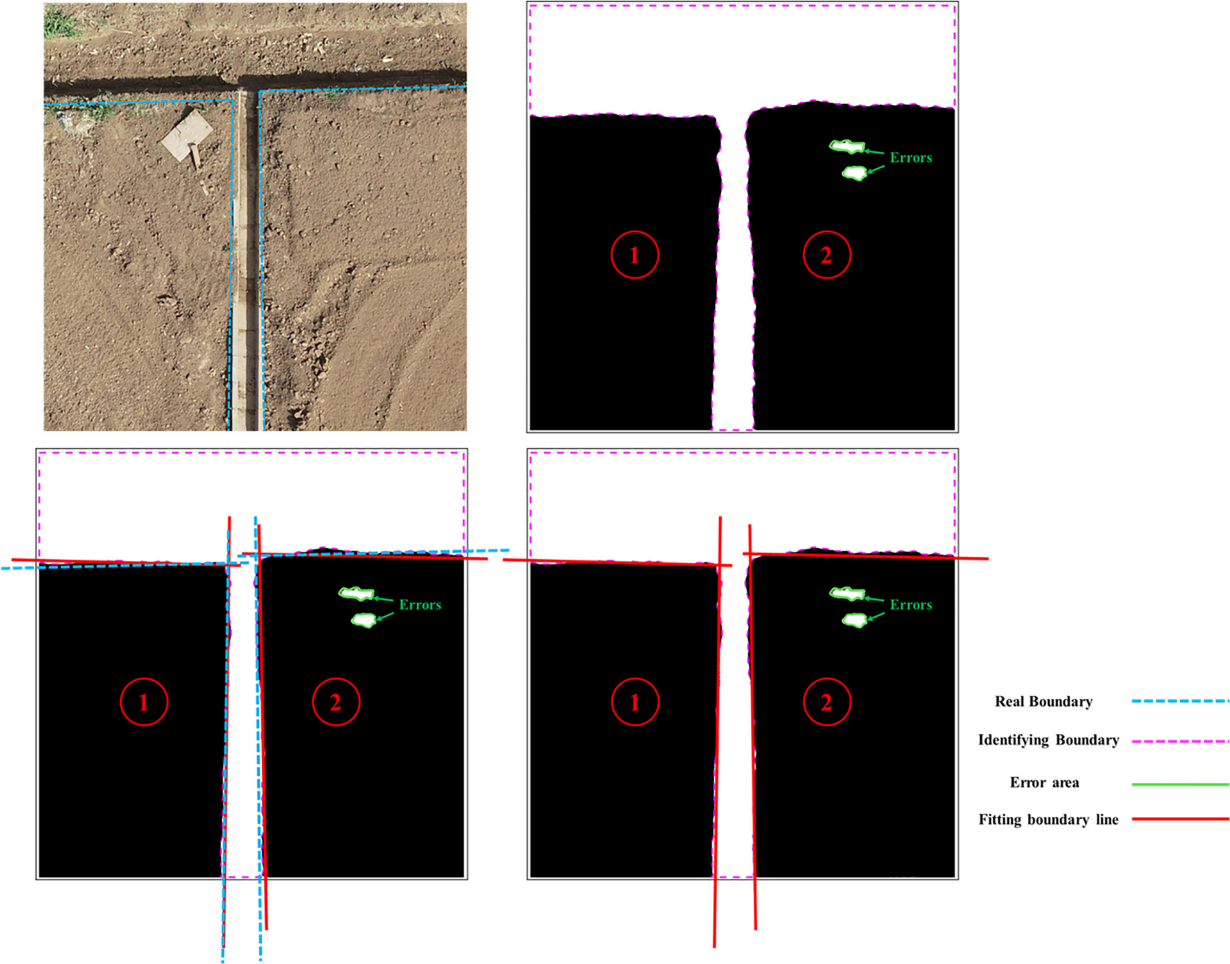
Boundary line fitting process (with multi-directional boundaries as an example). The red dashed line is the true boundary, the purple dashed line is the segmentation result boundary, and the blue dashed line is the fitted boundary.

Based on the results from *Section 2.3.1*, **Figure 8** illustrates the entire boundary line fitting process. The first step is to detect the number of boundary lines within a single region (① or ②). By examining the obtained boundary coordinate set, the orientation of the boundary lines in the image is determined (horizontal or vertical). If there is a significant variation in the *x*-values of some points in the set, accompanied by a small variation in the *y*-values, it is considered a horizontal boundary. In this case, points with *y*_Δ_ =|*y_i_* - *y_i_*_+1_|ϵ[0,50] are classified as points on the horizontal boundary line, forming the horizontal boundary point set. The same approach applies to identifying vertical boundary lines.

To mitigate the influence of inaccurate boundary segmentation in the segmented image on the fitting results of the LS algorithm, this study utilizes standardized residuals to identify and remove outliers in the boundary point set *P*. This approach effectively improves the fitting accuracy of the LS algorithm on the boundary and reduces the impact of outliers on the fitting results. The formula for calculating the standardized residual is shown as Equation (7):


(7) $${\text{Z}}_{\text{ei}}=\left|\frac{{\text{e}}_{\text{i}}}{{\text{S}}_{\text{e}}}\right|=\left|\frac{{\text{y}}_{\text{i}}-{\hat{\text{y}}}_{\text{i}}}{{\text{S}}_{\text{e}}}\right|$$

In this context, *y_i_* represents the observed value of the dependent variable, and ${\hat{y}}_{i}$ is the predicted value obtained from the estimated regression equation, while *e_i_* is the residual of the *i*th observation. We establish a rule that if the standardized residual *Z_ei_* > 3, that point is considered an outlier and is therefore removed. Subsequently, each data point within the transverse boundary point set *P* is defined as *P_n_* (*x_n_*,*y_n_*), and we conduct a polynomial linear regression using the method of least squares. The data points in this set all originate from multiple samplings of Equation (8).


(8) $$f\left(x_{i}\right)={\text{θ}}_{0}+{\text{θ}}_{1}x_{i}+{\text{θ}}_{2}x_{i}^{2}+\cdots +{\text{θ}}_{n}x_{i}^{n}$$

In the formula, *n* represents the order of the polynomial, and θ represents the coefficients of each term of the polynomial.

At this point, the sum of the squared errors for each data point within the dataset *P* is represented as shown in Equation (9).


(9) $$S=\sum_{i=1}^{m}{\left[f\left(x_{i}\right)-y_{i}\right]}^{2}$$

The function *f*(*x_i_*) is the result obtained as per Equation (8). Finally, all the boundary lines of this plot are summarized, followed by the boundary coordinate screening and boundary line fitting for other plot areas, until all the boundary lines of the image are completely processed.

#### 2.3.3 Boundary line evaluation indicators

**Figure 9:**
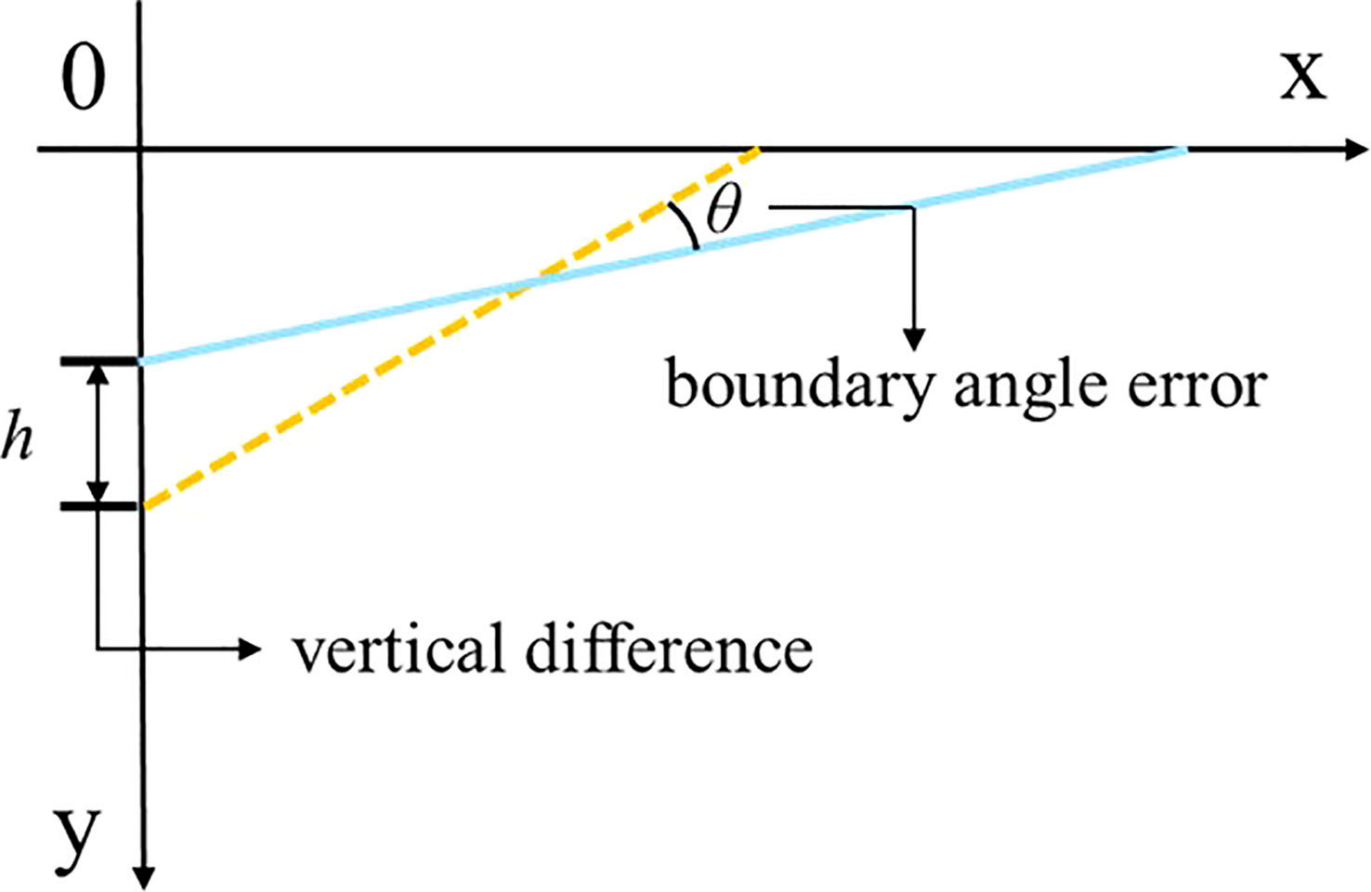
Schematic diagram of angular and vertical errors. The yellow dashed line is the real farmland boundary line and the blue solid line is the predicted farmland boundary line.


In this study, in order to measure the error between the fitted boundary line and the actual boundary of the farmland, we establish a Cartesian coordinate system as shown in **Figure 7F** of *Section 2.3.1*. We introduce the angular error and the vertical error as the standards for evaluating the accuracy of the boundary line extraction (He et al., 2022), as shown in **Figure 9**. The angular error is the angle between the actual boundary line and the fitted boundary line. When the actual boundary line and the predicted boundary line are in the same right angle coordinate system, the absolute value of the difference between the vertical coordinates when the horizontal coordinate is 0 is called the vertical difference, and the ratio of the vertical difference to the total pixel height of the image is the vertical error defined in this paper, and the calculation formula is shown as in Equation (10).

(10) $$\text{Vertical error}=\frac{\left|\text{vertical difference}\right|}{\text{Height of the image}}$$

## 3 Results and discussion

In order to verify the effectiveness of the method, ablation experiments and comparative tests on the segmentation network are conducted in this paper, and the final boundary extraction results are evaluated. During the experimental process, all other conditions are kept unchanged unless otherwise specified, and the experimental conditions and related parameter configurations are kept the same as in *Section 2.2.2*.* A* total of 130 images were included in this test, none of which were involved in training.

### 3.1 Ablation study and result analysis

#### 3.1.1 Ablation on attention mechanism

The attention mechanism module can enhance the adaptability of the encoder–decoder structured network, improve the feature effect of the encoder output, and thus increase the accuracy of the segmentation results. By carrying out ablation experiments on the attention mechanism, we compare CBAM with two other commonly used attention mechanisms, SENet [Squeeze-and-Excitation Network (Hu et al., 2018)] and ECANet [Efficient Channel Attention Network (Wang et al., 2020)]. In conditions where other configurations and parameters remain the same, the attention mechanisms are added at the same location. The results are shown in **Table 1**. The mIoU of Experiment 1 is 0.48% and 1.87% higher than Experiments 2 and 3, respectively. The accuracy of Experiment 1 is 0.31% and 0.82% higher than Experiments 2 and 3, respectively. In summary, while CBAM theoretically has a higher recognition accuracy, because it combines two kinds of attention mechanisms, its theoretical recognition speed is slower compared to the other two mechanisms. Adding the CBAM module to MobileNetV2 can effectively enhance the accuracy of the network in UAV remote sensing image recognition tasks, thereby increasing the network’s predicted PA and mIoU.

**Table 1:** In the same configuration and parameters, Experiments 1–3 are the results of adding CBAM, SENet, and ECANet to the same location, respectively.

Test Num	CBAM	SENet	ECANet	mIoU (%)	Accuracy (%)	Reasoning time (ms)
**1**	**√**	**-**	**-**	**87.67**	**95.17**	**0.081**
2	–	√	–	86.74	94.86	0.076
3	–	–	√	85.35	94.35	0.074

Bold values represents the modules or models used in this study.

#### 3.1.2 Impact of different model improvement options on performance

To validate the effectiveness of the various improvements in AttMobile-DeeplabV3+, we conducted a comparative analysis before and after the network modification. As shown in **Table 2**, the analysis results indicate that AttMobile-DeeplabV3+ performs best in all indicators, with mIoU, mPA, and reasoning time being 88.98%, 94.05%, and 0.080 ms, respectively. DeeplabV3+(MobileNetV2) is the simplest network among the four groups of experiments, with the lowest complexity, but the worst data in all indicators. DeeplabV3+(Attention-MobileNetV2) achieves significant improvements in mIoU and mPA by adding an attention mechanism, but its reasoning time slightly increases compared to DeeplabV3+(MobileNetV2). Experiment 3 slightly improves in terms of mIoU and mPA, but the increase in reasoning time is considerable. In summary, the direction of improvement for DeeplabV3+ is correct, AttMobile-DeeplabV3+ has the best overall performance, but its complexity is relatively high.

**Table 2:** Performance comparison before and after.

Number	Name	Complexity (M)	mIoU (%)	mPA (%)	Reasoning time (ms)
1	MobileNetV2	5.814	85.90	92.15	0.075
2	Attention-MobileNetV2	5.839	87.67	93.36	0.081
3	ASPP only improvement	6.044	86.99	92.57	0.078
**4**	**AttMobile-DeeplabV3+**	**6.070**	**88.98**	**94.05**	**0.080**

Experiments 1–4 correspond to the following models: DeeplabV3+ with MobileNetV2 as backbone network, DeeplabV3+ with Attention-MobileNetV2 as backbone network, DeeplabV3+ with only improved ASPP module, and AttMobile-DeeplabV3+. Bold values represents the modules or models used in this study.

**Figure 10:**
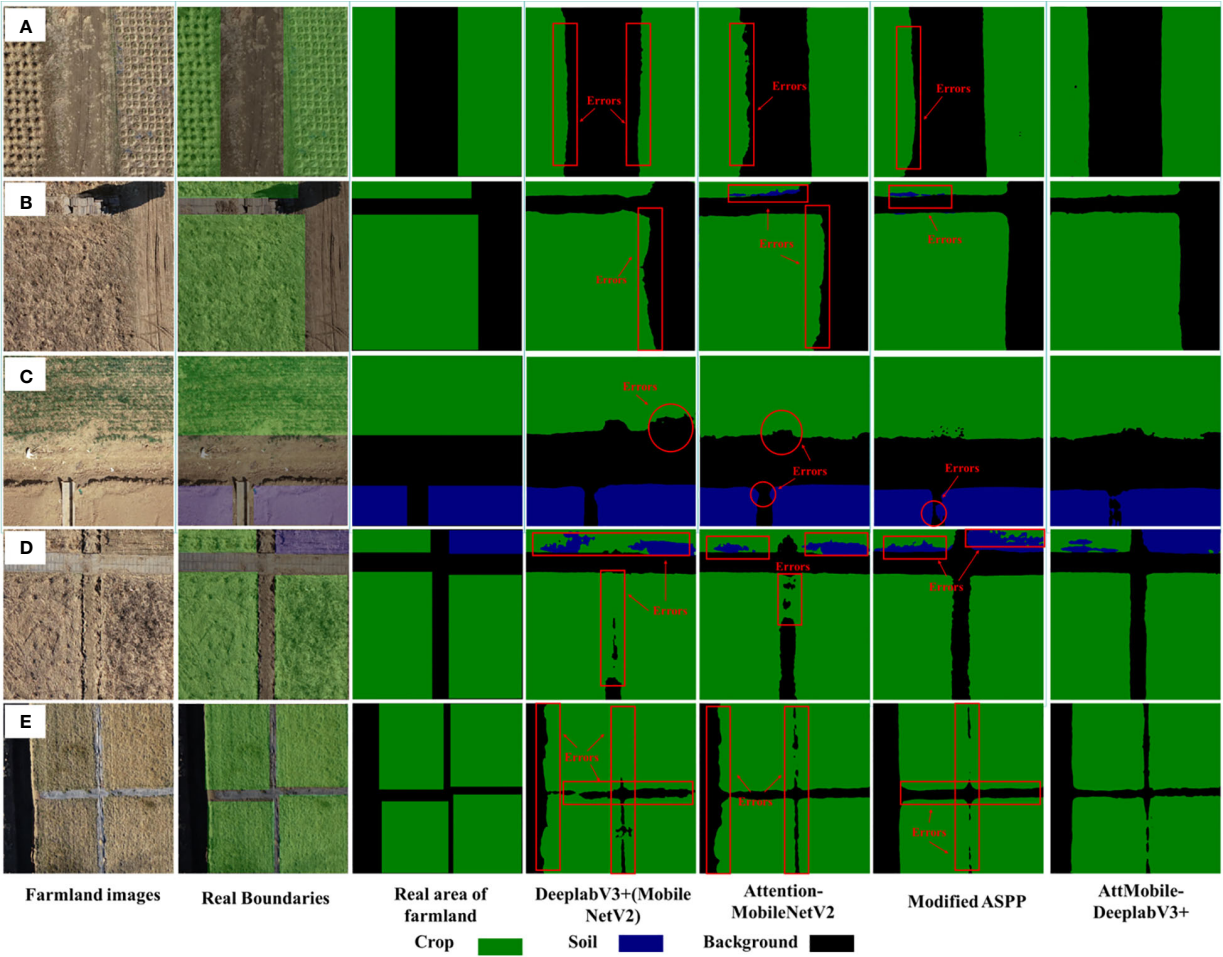
**(A–E)** Comparison of image recognition results before and after model modification.

The farmland images are divided into two main categories (image a contains the background and one of the labels, and image b contains the background and two labels), and the images in a are further divided into those where the farmland boundaries appear parallel in the image and those where the farmland boundaries appear interlaced in the image. Therefore, to further verify the impact of the improvements on the image segmentation effect, multiple farmland images of three different classification cases are selected for illustration in this paper, as shown in **Figure 10**. The red markers highlight the locations where segmentation errors occurred. From the overall effect of multiple images, it can be seen that DeeplabV3+ (with MobileNetV2 as the backbone network) can recognize feature information correctly. However, in **Figures 10A, B, E**, this network’s recognition of the long straight boundaries of the farmland is poor, and in **Figure 10C**, there is a phenomenon of missing farmland. Also, in **Figure 10D**, the vertical boundary is largely unrecognizable. After changing the backbone network to Attention-MobileNetV2, the network can better recognize the farmland covered by crops, offering more details. Still, the recognition of long straight plot boundaries is not accurate enough, and there are cases of part misclassification, which have been marked. By keeping the backbone network as MobileNetV2 and adding extra pooling layers in ASPP, the network’s recognition accuracy for long straight plot boundaries has been greatly improved.

In conclusion, the direction of improvement for AttMobile-DeeplabV3+ is validated. Specifically, using Attention-MobileNetV2 as the backbone network can achieve a lightweight balance between speed and accuracy. The improvements to the ASPP module can optimize the recognition tasks of boundaries with long-distance dependencies, significantly enhancing recognition results. Compared to the control experiment, the AttMobile-DeeplabV3+ network that combines all improvement proposals is more accurate and complete in boundary segmentation in agricultural environments, whether in terms of detail handling or accuracy in recognizing long straight plot boundaries.

### 3.2 Effective results of AttMobile-DeeplabV3+

To validate the effectiveness of the AttMobile-DeeplabV3+ network in obtaining boundary information of farmland from drone remote sensing images, we conducted comparative experiments with several commonly used semantic segmentation networks, including U-Net, DeeplabV3+ (ResNet50), BiSeNetV2, and HR-Net. The values of various evaluation indicators are shown in **Table 3**, which respectively display the mIoU, mPA, and IoU value of each label for each network. The theoretical values of DeeplabV3+ (ResNet50) perform well, while BiSeNetV2 and HR-Net perform somewhat worse across all categories, although they still yield respectable results. U-Net has the lowest mIoU among all networks at 82.03%, making it the worst in terms of overall segmentation theoretical values. AttMobile-DeeplabV3+ achieves the highest evaluation index theoretical values among all networks, outperforming the control group networks. Its mIoU is 6.25%, 4.89%, 3.18%, and 0.45% higher than that of U-Net, HR-Net, BiSeNetV2, and DeeplabV3+ (ResNet50), respectively.

**Table 3:** Performance comparison of different models (%).

Model	mIoU	mPA	Crop (IoU)	Soil (IoU)	Background (IoU)
U-Net	82.03	92.43	89.40	92.01	66.48
DeepLabv3+(ResNet50)	88.53	93.97	93.16	92.33	80.11
BiSeNetV2	85.80	92.20	92.52	91.48	73.39
HR-Net	84.09	92.57	90.41	89.85	72.03
**AttMobile-DeeplabV3+**	**88.98**	**94.05**	**93.25**	**93.14**	**80.54**

Bold values represents the modules or models used in this study.

**Figure 11:**
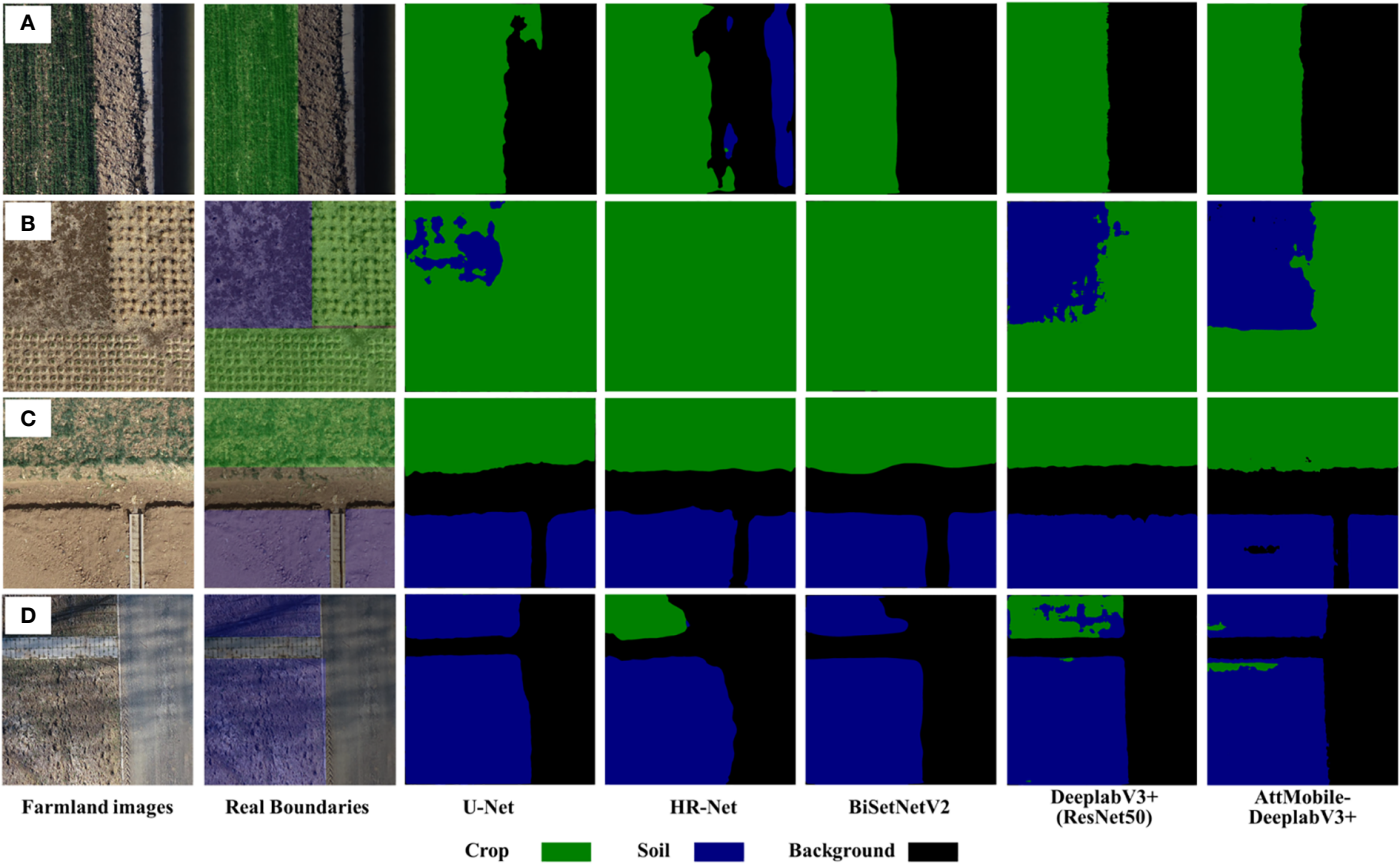
**(A–D)** Analysis of comparative test results of different models.

The segmentation results of each network on the test set are shown in **Figure 11**. Analyzing the recognition results of **Table 3** and **Figure 11**, all comparison networks have issues with insufficient recognition, and some networks also have misclassification issues. Overall, the AttMobile-DeeplabV3+ network has the best recognition effect, its segmentation of farmland boundary is smoother, and its recognition of long straight lines is more accurate and complete, which provides a good basis for the subsequent research on boundary line extraction.

### 3.3 Analysis of boundary line extraction results

In order to verify the accuracy of this algorithm in different farmland boundary extraction tasks, three representative images (a), (c), and (d) were selected from **Figure 11**. Boundary fitting verification was conducted for all boundaries in the images, and the error analysis results are shown in **Table 4**. The average vertical error of fitting the Crop boundary line is 0.038, and the average angular error is 1.976°, while the vertical error of fitting the Soil boundary line is 0.040, and the average angular error is 1.347°. The overall average vertical error of the fitted boundary line is 0.039, and the average angular error is 1.473°. These results meet the accuracy requirements for farmland boundaries of unmanned agricultural machinery and provide a data basis for subsequent boundary coordinate extraction.

**Table 4:** Error analysis of fitting a boundary line using the fitting algorithm.

Evaluation indicators	Crop	Soil	Mean
Vertical error	0.038	0.040	0.039
Angular error (°)	1.976	1.347	1.473

3 decimal places reserved.

## 4 Conclusion

This study proposes a semantic segmentation network based on DeeplabV3+, and designs a method to extract farmland boundaries from UAV remote sensing images using this network. The principles and structure of this method are analyzed, and ablation experiments and comparative experiments are conducted. On the crop-covered farmland and the uncropped farmland, the IoU of the network is 93.25% and 93.14%, respectively, and the PA of the crop-covered farmland is 96.62%. The segmentation results show that the AttMobile-DeeplabV3+ can accurately identify crop-covered farmland and uncropped farmland. The proposed method significantly improves the accuracy of farmland boundary positioning and the ability to identify boundary details, effectively solving the problem of incomplete boundary segmentation in the UAV farmland image recognition method. At the same time, the results of the boundary line fitting were also evaluated, with an average vertical error and average angular error of 0.039 and 1.473°, respectively. The results of boundary line fitting show that the proposed method has high accuracy, and can provide practical and accurate method support for subsequent autonomous unmanned agricultural machinery for farmland boundaries. At the same time, this method is expected to be widely applied in different types of farmland, providing a practical method for the current acquisition of farmland boundary information.

However, there are some limitations to this research. Because of factors such as lighting, the shadows of obstacles in the farmland cannot be completely eliminated, which, to some extent, affects the extraction accuracy of the farmland boundaries and interferes with the accuracy of boundary line identification. Therefore, in future work, we need to further improve the semantic segmentation network and equip it with more advanced training platforms and clearer image collection equipment to solve the existing problems and achieve the long-term goal of precision agriculture.

## Data availability statement

The raw data supporting the conclusions of this article will be made available by the authors, without undue reservation.

## Author contributions

HL and HW designed the study. HL, HW, ZJM, ZFM, YR, WF, SH and GZ performed the experiments. HL, HW and ZJM analyzed the data. HL, HW, YS and ZJM discussed the data and wrote the manuscript. All authors contributed to the article and approved the submitted version.
